# Plant sterols lower LDL-cholesterol and triglycerides in dyslipidemic individuals with or at risk of developing type 2 diabetes; a randomized, double-blind, placebo-controlled study

**DOI:** 10.1038/s41387-018-0039-8

**Published:** 2018-05-25

**Authors:** Elke A. Trautwein, Wieneke P. Koppenol, Arienne de Jong, Harry Hiemstra, Mario A. Vermeer, Manny Noakes, Natalie D. Luscombe-Marsh

**Affiliations:** 1 Unilever R&D, Vlaardingen, The Netherlands; 2 Commonwealth Scientific and Industrial Research Organisation (CSIRO), Health and Biosecurity, Adelaide, Australia

## Abstract

**Background:**

Managing cardiovascular disease (CVD) risk factors, e.g., dyslipidemia in type-2 diabetes mellitus (T2DM) is critically important as CVD is the most common cause of death in T2DM patients. This study aimed to investigate the effect of plant sterols (PS) on lowering both elevated low-density lipoprotein cholesterol (LDL-C) and triglycerides (TG).

**Methods:**

In a double-blind, randomized, placebo-controlled, parallel study, 161 individuals at increased risk of and with established T2DM, consumed low-fat spreads without or with added PS (2 g/d) for 6 weeks after a 2-week run-in period. Increased risk of developing T2DM was defined by the Australian T2DM Risk Assessment Tool (AUSDRISK). Fasting serum/plasma total cholesterol (TC), LDL-C, TG, high-density lipoprotein cholesterol (HDL-C), glucose and insulin were measured at baseline and after 6 weeks. Effects on acute and chronic postprandial blood lipids, glucose and insulin were measured over 4-h in 39 individuals with T2DM following a mixed meal challenge without and with added 2 g/d PS at week 6. The study was registered at clinicaltrials.gov (NCT02288585).

**Results:**

Hundred fifty-one individuals completed the study and 138 (57% men, 43% women; 44 with and 94 at risk of T2DM) were included in per protocol analysis. Baseline LDL-C and TG were 3.8 ± 1.0 and 2.5 ± 0.8 mmol/l, respectively. PS intake significantly lowered fasting LDL-C (−4.6%, 95%CI −1.2; −8.0; *p* = 0.009), TC (−4.2%, 95%CI −1.2; −7.1; *p* = 0.006) and TG (−8.3%, 95% −1.1, −15.0; *p* = 0.024) with no significant changes in HDL-C, glucose or insulin. Postprandial lipid (TG, TC, LDL-C, HDL-C, remnant cholesterol), glucose and insulin responses did not differ.

**Conclusions:**

In individuals at risk of and with established T2DM and with elevated TG and LDL-C, 2 g/d of PS results in dual LDL-C plus TG lowering. Postprandial lipid or glycemic responses did not differ between PS and control treatment.

## Introduction

Type 2 diabetes mellitus (T2DM) is an independent risk factor for cardiovascular disease (CVD) and individuals with T2DM have on average a 2-fold increase in CVD risk compared to those without diabetes^1–3^. Managing CVD risk factors such as dyslipidemia is critically important as CVD is the major cause of death in T2DM patients. Many individuals at risk of developing or with T2DM have dyslipidemia characterized by lipid and lipoprotein abnormalities such as elevated fasting and non-fasting triglycerides (TG) and TG-rich lipoproteins, e.g., chylomicrons and very-low-density lipoprotein (VLDL), low high-density lipoprotein cholesterol (HDL-C) and often also elevated low-density lipoprotein cholesterol (LDL-C) concentrations, in the form of increased small dense LDL particles^2,4^. The underlying abnormalities in postprandial and fasting lipid and lipoprotein metabolism contributing to diabetic dyslipidemia are beginning to be understood such as reduced hepatic uptake of chylomicrons and their remnants and impaired VLDL secretion^4^. Furthermore, insulin resistance in adipose tissue contributes to diabetic dyslipidemia by failure to suppress lipolysis^5^.

Lowering LDL-C below 2.6 mmol/l is the primary target of blood lipid lowering therapy in T2DM. For that reason, individuals with established T2DM are advised to use statin therapy to control elevated LDL-C and reduce cardiovascular risk^6^. An additional lowering of elevated TG concentrations can further contribute to reducing residual CVD risk. A healthy diet pattern can also help to improve diabetic dyslipidemia and control elevated total cholesterol (TC), LDL-C and, TG concentrations as is emphasized in clinical guidelines^7^.

Amongst dietary strategies to control blood lipids, plant sterols (PS) or stanols have been shown to lower LDL-C concentrations by comparable magnitudes in individuals with T2DM as in hypercholesterolemic, but otherwise healthy people^8–11^. A meta-analysis of randomized, placebo-controlled studies with patients diagnosed with T2DM concluded that PS or stanols at intakes of 1.6–3.0 g/d significantly lowered TC and LDL-C concentrations by 0.26 and 0.31 mmol/L, respectively^8^. While the LDL-C lowering effect of PS and stanols in individuals with T2DM has been established, evidence for an additional TG lowering effect is still scarce. Available evidence is based on studies that primarily investigated the LDL-C lowering effect of PS or stanols, while a TG effect was always a secondary study outcome and therefore may be underpowered to detect a significant lowering of TG concentrations. Previous meta-analysis pooling data from 12 studies found a significant 6% TG lowering effect with a PS intake ranging between 1.6 and 2.5 g/d; this effect was more pronounced in individuals with higher baseline TG concentrations^12^. Similarly, a TG lowering effect of plant stanols, with the largest reductions seen in individuals with higher baseline TG concentrations, was also reported in the meta-analysis of Nauman et al.^13^. However, a TG lowering benefit of PS or stanols has so far not been explicitly studied in individuals at risk or with established T2DM. The meta-analysis of Baker et al.^8^ found no apparent effect of PS on TG concentrations among patients with T2DM, which is possibly related to the small number of participants in the limited number of studies. Furthermore, many previous studies excluded individuals with high basal TG and therefore a TG-lowering benefit of PS and stanols may have been overlooked.

Summarizing the available study evidence, Plat et al.^10^ concluded that in T2DM patients with relatively low TG levels, TG were lowered by 0.06 mmol/l or 3.7% with an average PS/stanol intake of 1.85 g/d. Studies enrolling individuals with the metabolic syndrome who had basal TG concentrations exceeding 1.7 mmol/l found however a TG lowering effect of 0.66 mmol/l or 13.9% with an average intake of 3.2 g/d of PS/stanols^10^.

As individuals with T2DM or at risk of developing T2DM are at increased risk for CVD and often have elevated TG and LDL-C, PS could lead to a potential dual blood lipid benefit in lowering both LDL-C and TG in these populations. So far, studies explicitly investigating such a dual lipid lowering effect of PS have not been performed in these populations.

Moreover, the capacity of PS/stanols to attenuate the known atherogenicity of postprandial lipids and glucose increases remains unclear. It has recently been voiced that there is a need for studies exploring the effects of acute and chronic PS or stanol intake in affecting postprandial response in populations at risk of developing T2DM or with established T2DM^14^. Two recent studies that measured postprandial TG concentrations after a meal challenge including added PS or stanols showed no effect on postprandial TG concentrations during 4 to 8 h in healthy individuals^15,16^. A limitation of these studies is that study participants were not pre-selected based on their baseline TG concentrations, which were consequently in a healthy range of 1.1 to 1.3 mmol/l.

The aim of this study was to investigate the effect of PS intake on fasting and acute as well as chronic postprandial blood lipids (TG, TC, LDL-C, HDL-C) in individuals at risk and with established T2DM. To our knowledge this is the first study to investigate a dual blood lipid lowering benefit, i.e., lowering of fasting LDL-C and TG of PS in this study population. Also, the effects on fasting and postprandial blood glucose and insulin were explored.

## Materials and methods

This study took place from November 2014 to September 2016 at the Commonwealth Scientific and Industrial Research Organisation (CSIRO), Food and Nutrition group, in Adelaide and Sydney, Australia. The study was conducted in accordance with applicable laws and regulations including, the International Conference on Harmonization (ICH), Guideline for Good Clinical Practice (GCP) and the ethical principles that have their origins in the Declaration of Helsinki. The protocol, informed consent, advertisements, and protocol amendments were approved by the CSIRO Food and Nutrition Flagship Human Research ethics committee. Written informed consent was obtained from all study participants. The study was registered at clinicaltrials.gov as NCT01803178.

### Study design

The study was designed as a randomized, double-blind, placebo-controlled, parallel study with two intervention arms: a low-fat spread (40% fat) with 11% added PS in the form of PS esters and a control spread without added PS. The intervention period lasted 6 weeks and was preceded by a run-in period of 2 weeks during which all study participants consumed the placebo spread allowing to get familiarized with consuming the study products and for blood lipid concentrations to stabilize. At the end of the run-in (i.e., baseline) and intervention phases, fasted blood samples were drawn on two consecutive days for measuring serum lipid, insulin, and plasma glucose concentrations. On all test days, participants came to the study center in a fasted state (12 h of neither food nor drinks except water) and received breakfast after all measurements were performed.

At the end of the intervention after 6 weeks, postprandial blood lipids, glucose and insulin were measured over 4 h after a mixed meal in a randomly selected sub-group of study participants all with established T2DM. The test meal consisted of a high-fat drink with 240 ml fresh milk plus 85 g Scandishake® and 40 ml Calogen Neutral® drink (both from Nutricia, North Ryde, Australia) plus two slices of white bread with 20 g strawberry jam and 20 g spread without added (placebo) and with 2 g PS. The nutrient composition of the test meal was as follows: 4232 kJ (1001 kcal) energy, 50% from fat, 43% from carbohydrate, and 7% from protein. The total fat content was 58 g of which 19 g were saturated fatty acids (SAFA), 12 g polyunsaturated fatty acids (PUFA), 25 g monounsaturated fatty acids (MUFA). The test meal was prepared by the research dietitian on the morning of the test day. For the subgroups who underwent the mixed meal challenge, 50% of those who received the PS-added spread during the 6 weeks of intervention, were challenged with the PS-added spread and the other 50% were challenged with the placebo spread. For those who received the placebo spread during the 6-week intervention, 50% were challenged with the PS-added spread and the other 50% with the placebo spread. In this way, short (acute), longer-term (chronic), and acute upon chronic effects of PS were studied.

Health and wellbeing, use of concomitant medication, and adverse events (AEs) were monitored throughout the study.

### Study population

The study population consisted of individuals with established and controlled T2DM on a stable dose of glucose lowering or statin medication for ≥3 months and individuals at high risk of developing T2DM as assessed by the validated Australian T2DM Risk Assessment Tool AUSDRISK; score of ≥12 using an online tool http://www.health.gov.au/preventionoftype2diabetes. The AUSDRISK tool is a simple risk score predicting diabetes risk based on demographic, lifestyle, and simple anthropometric information^17^.

Participants were invited by invitation letter sent to eligible volunteers registered on the CSIRO participant database or to people listed with two external clinical trials recruitment agencies, by public advertisements using radio, television, print media, and various social media platforms like Facebook, as well as online newsletters and notice boards in general practitioner or specialist endocrinologist/cardiology and research clinics located within metropolitan Adelaide and Sydney, Australia.

After giving informed consent and being pre-screened, potential participants were asked to complete a medical screening questionnaire collecting information about medical history and medication usage, and the risk of developing T2DM. Based on the pre-screening response, individuals who met the age criteria and did not report any information that excluded them from the study, were invited to attend an in-person full-screening session following an overnight fast. At this full-screening visit, height, body weight, waist circumference, blood pressure, and heart rate were measured. In addition, a spot urine sample was collected for assessment of albumin-to-creatinine ratio, and a blood sample for assessment of clinical chemistry and hematology, HbA1c, and fasting blood lipid concentrations. Participants were eligible to be enrolled in the study when they met the following main predefined inclusion and exclusion criteria: aged 30–75 years, having a BMI > 20.0 kg/m^2^, TG > 150 mg/dl or 1.74 mmol/l, HbA1C ≤ 8.5% or ≤69 mmol/mol if they had established T2DM, or <6.50% or <48 mmol/mol if they were at high risk for developing T2DM, had blood pressure, heart rate, hematological, and clinical chemical parameters within the normal reference, were willing to comply with the test product intake and the dietary restrictions of the study, agreed to be informed about medically relevant personal test results by a physician and had signed the informed consent. Participants were excluded if they had been recently (within 1 year) diagnosed with cardiovascular event(s), systemic inflammatory conditions, had a medical condition which might impact study measurements, used PS or stanol-enriched foods or supplements in the 3 months prior to the screening and/or during the study, used oral antibiotics (except for topical antibiotics) in 40 d or less prior to screening, used over-the-counter and prescribed medication which might interfere with study measurements (e.g., Ezetimibe, fibrates, niacin, fish oil, fast acting insulin or oral antibiotics), used a high dose statin treatment other than being on a stable and low dose for ≥3 months, were pregnant or lactating; reported alcohol consumption of >14 standard drinks or unwilling to reduce intake prior to screening or for study duration, reported intense sporting activities of >10 h/week, reported weight loss or gain of 3 kg or more during a period of 2 months prior to screening, were an employee of Unilever or CSIRO’s Clinical Research Unit, were currently smoking or being a non-smoker for less than 6 months and reported use of any nicotine containing products in the 6 months prior to screening and/or during the study or had a(n) (known) allergy/intolerance to the study products.

Eligible participants were randomized to either the PS-added margarine or the placebo margarine group using a permuted block randomization stratification process to ensure that each treatment had approximately balanced numbers with respect to diabetes status (at risk or with established T2DM), gender (male/female), age (30–50 years or 51–75 years), and screening LDL-C (those at-risk of T2DM: 2.95–3.94 mmol/l and 3.95–4.94 mmol/l or those with established T2DM: 2.15–3.54 mmol/l and 3.55–4.94 mmol/l).

### Study intervention and products

During the run-in period, participants were provided with the placebo spread and during the intervention period, participants were provided with the spread with added PS or the placebo spread. Each day, participants consumed two 10-g portions with main meals. The PS-added spread contained 18.5% PS esters, equivalent to 11.1% free PS, i.e., 2.2 g PS in 20 g margarine). All spreads were produced by Unilever at a factory site in Pratau, Germany; PS esters were sourced from BASF, Germany. The formulations of the two spreads were kept similar, except for the free PS replacing water in the recipe. The total fat content of the spreads was 40%. The contents of SAFA, MUFA, and PUFA were similar in both spreads with per total fat 20–22% SAFA, 23% MUFA, and 53–55% PUFA. The test products were matched regarding taste and appearance. The nutritional composition of the study products is summarized in Table 1. Concentrations of PS were measured in a random selection of study spreads across all production batches; the amount of PS in the PS-added was on average 11.3%, resulting in a PS intake of 2.26 g per 20 g daily margarine consumption.

**Table 1:** Nutritional composition of the study spreads

Nutritional composition per 100 g margarine	Control spread	PS-added spread
Energy, kJ (kcal)	1479 (360)	1481 (360)
Total protein, g	0.0	0.0
Total carbohydrates, g	0.0	0.0
Total fat, g	40.0	40.0
SAFA, g	8.21	8.93
MUFA, g	9.34	9.07
PUFA, g	22.00	21.38
Total n-6 PUFA, g	17.70	17.32
Total, n-3 PUFA, g	4.30	4.06
Trans FA, g	0.60	0.63
Cholesterol, mg	0.80	0.66
PS ester^a^, g	0.0^b^	18.5^b^
Sodium, mg	9.10	7.43
Vitamin A, μg	825.0	799.2
Vitamin D3, μg	7.50	7.50
Vitamin E, mg	20.42	19.48
Fiber, g	0.0	0.0
Water, g	60.0	48.9

*FA* fatty acids, *SAFA* saturated FA, *MUFA* monounsaturated FA, *PUFA* polyunsaturated FA

^a^The PS mixture contained 70% sitosterol, 14% campesterol, 8% sitostanol, 3% brassicasterol and other minor plant sterols

^b^This equals 11.1 g free plant sterol equivalents

All study products were provided as 10-g portion packs. Participants were asked to refrigerate the spreads at home. Freezing was not acceptable and cooking, baking or frying with the spreads was not allowed; using the spread on top of hot dishes was acceptable and participants were instructed to add it to a meal when on the plate. Participants returned all opened and unopened spread tubs to the study center for a compliance check. Noncompliance with study product intake was defined as having consumed <90% of spread intake. Throughout the study, participants were asked to minimize changes in their habitual diet and lifestyle.

### Outcomes

The primary outcome measures were fasting serum LDL-C and TG concentrations, secondary outcomes included fasting TC, HDL-C, lipoprotein (a) (Lp(a)) and safety and tolerance of the study products and tertiary (exploratory) outcomes were fasting non-HDL-C, remnant cholesterol (remnant C), glucose and insulin, and postprandial TG, TC, LDL-C, HDL-C, glucose and insulin response after a mixed meal challenge.

### Study measurements

Throughout the study plasma/serum was prepared by centrifuging at 3000 rpm for 10 min at 4 °C (using a GS-6R centrifuge; Beckman, Fullerton, CA, USA). The resulting plasma/serum was stored at −20 °C until analyzed at the completion of the study. All samples from each subject were analyzed within the same analytic run. Samples were analyzed in two batches.

Fasting blood lipids were measured in two consecutive blood samples at baseline (day 1, 2) and end of the 6-week intervention (day 43, 44). Postprandial blood lipids were measured at the end of intervention (day 44) at timepoints −15 min, −5 min and +15 min, +30 min, +45 min, +60 min, +90 min, +120 min, +180 min and +240 min after the mixed meal challenge. Serum TC, LDL-C and HDL-C and TG, and plasma glucose concentrations, were measured using commercial enzymatic kits (Roche Diagnostics, Basel, Switzerland) on a Hitachi 902 auto-analyzer (Roche Diagnostics, Indianapolis, IN). Plasma Lp(a) concentrations were measured using a commercial kit (Randox Pty Ltd, Parramatta, NSW, Australia) on a Beckman AU480 auto-analyzer (Beckman, Fullerton, CA, USA). Serum insulin was measured using a commercial ELISA kit (Mercodia AB, Uppsala, Sweden). All blood measurements were carried out at CSIRO. Non-HDL-C (TC minus HDL-C) and remnant C defined as TC minus (HDL-C and LDL-C) were calculated.

### Statistical analysis and sample size calculation

Originally it was planned to recruit a total of 214 study participants. This study size calculation was based on the primary objective of the study, i.e., an effect of PS on blood TG concentrations. In total 194 subjects were needed (*n* = 97 per study arm), based on two TG measurements at baseline and after 6-week intervention, using a 2-sided alpha of 0.10, a power of 0.8 and an effect size equivalent to a 10% reduction in TG in the PS-added spread intervention group. The 10% reduction in TG was based on a baseline TG concentration of 2.3 mmol/l in individuals with T2DM as obtained from the literature. Considering a dropout rate of 10%, in total 214 subjects were required, 107 subjects per treatment group. The original study sample size could not be met due to limitations finding eligible study participants, esp. individuals with established T2DM even after intensified recruitment strategies. Hence, it was decided to reduce the number of study participants to 150 (*n* = 75 per study arm) to ensure the study was completed. It was re-calculated that a total of 150 completers based on two TG measurements at baseline and after 6-week intervention, using a 2-sided alpha of 0.10, would still provide a power of 0.8 and an effect size equivalent to detect a 11.5% reduction in TG concentration in the PS-added spread intervention group. The increase in lowering TG to 11.5% (instead of the original 10%) was justified based on recent findings^18^ and the fact that screening TG concentrations of participants already enrolled in the study (*n* = 126) at the time of revising the sample size was above 2.4 mmol/l.

The postprandial blood lipid and glucose/insulin response, was originally planned to be executed in a randomly selected subgroup of 46 individuals. Ultimately, 39 study participants, all with established T2DM, underwent the mixed meal challenge.

All statistical analyses were performed by Unilever R&D with the statistical software package SAS version 9.4 (SAS Institute Inc., Cary, NC, USA). Data were analyzed according to the intention-to-treat (ITT) and the per-protocol (PP) principle, i.e., excluding data from study participants who had been noncompliant with the protocol (i.e., low test product compliance, not being weight stable, or use of prohibited drugs). Here, the results based on the PP analysis are reported. Non-compliance was defined as having consumed less than 90% of total margarine intake, having gained or lost more than 5% of body weight, or not having been compliant with the diet and medication restrictions as judged by the study team during the blind review.

For TG, TC, LDL-C, HDL-C, non-HDL, remnant C, Lp(a), glucose and insulin, changes from baseline after intervention with PS were compared to placebo and analyzed using an ANCOVA model. The predefined, basic ANCOVA model used log (change from baseline) as response variable and contained log (baseline value) and treatment as predictors. Additional a priori predictors were added to this basic model, amongst others age, gender, site identifier (Adelaide or Sydney), risk-group (established T2DM or at risk of T2DM), and risk-group*treatment interaction. Bayesian Information Criterion (BIC) as a goodness-of-fit criterion was used to assess the contribution of these additional predictors in the model by exploring all possible combinations; more than 1000 models were generated by this approach. The results of the model with the smallest BIC, i.e., the “best” model was selected and is reported. Working on a log scale for the response allowed to represent the resulting treatment effect (LS-means) as a percentage change relative to the placebo treatment and an associated 95% confidence interval (CI).

The differences in shape between the postprandial curves for blood lipids, glucose and insulin response were explored using a repeated measures ANOVA model. The ANOVA model contained as predictors (all class variables): time, the 6-week intervention product (PS-added or placebo spread), type of meal challenge (PS-added or placebo spread), time*intervention product-interaction and the time*type of meal challenge-interaction. Other a priori predictors included in the model were e.g., gender and age; different covariance structures were also evaluated. A significant contribution of time*intervention product-interaction and/or time*type of meal challenge-interaction indicates a different shape of the response curve created by intervention product and/or type of meal challenge. A significant contribution of 6-week intervention product, type of meal challenge or any other predictor indicates a vertical shift of the curves while the shape remains the same. The contribution of the predictors in combination with covariance structure was assessed based on BIC and the results of the “best” model (smallest BIC) is reported. In addition, for post prandial curves of glucose the positive incremental area under the curve (piAUC) and for insulin the total area under the curve (tAUC) were calculated.

## Results

### Participant characteristics and dietary compliance

**Fig. 1:**
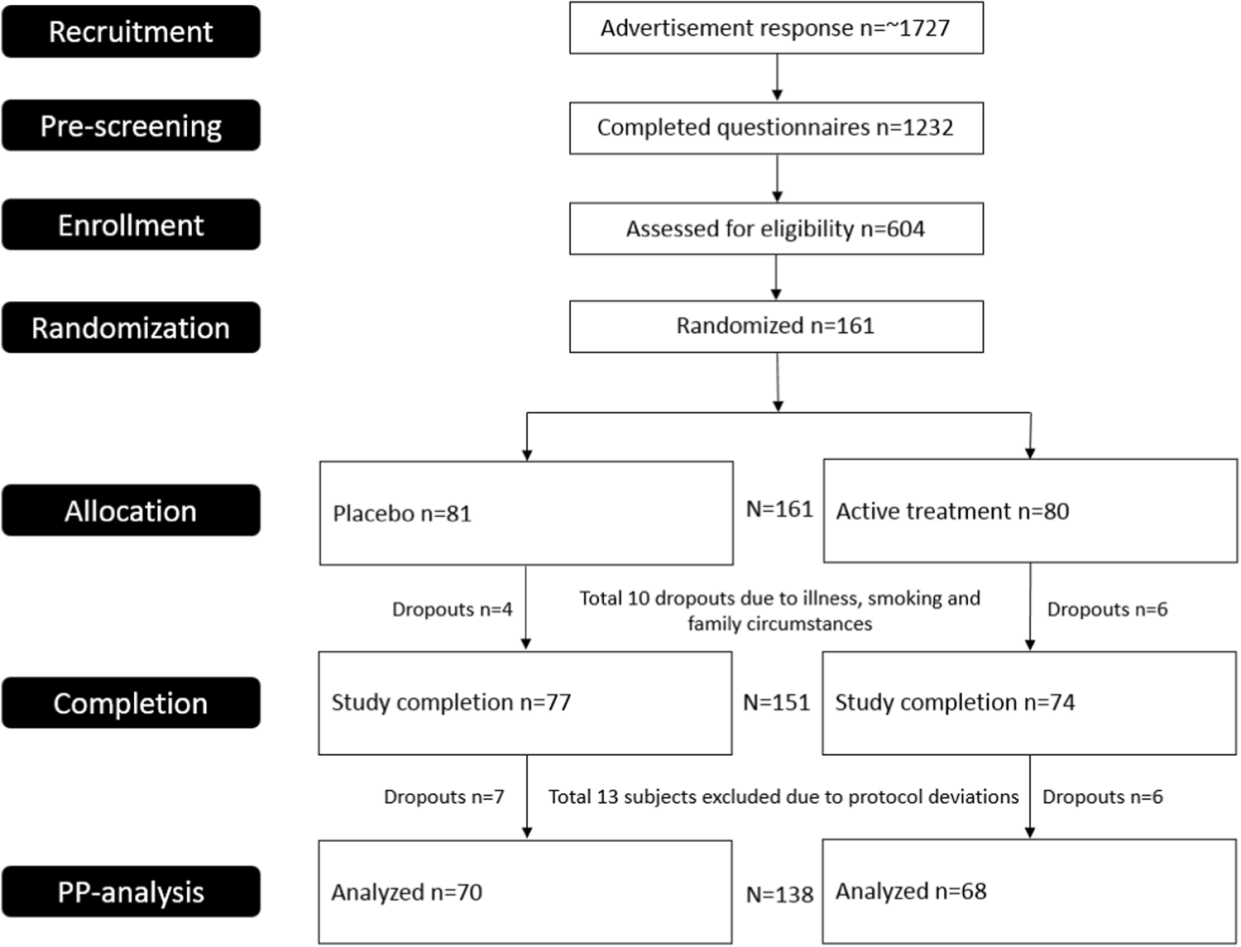
**Flow diagram depicting flow of participants throughout this randomized, double-blinded, placebo-controlled study on effects of plant sterols on blood lipids in dyslipidemic individuals with established and at risk of developing T2DM**

In total, 1232 individuals completed the pre-screening questionnaire. Of those, 604 completed the full screening and 161 were identified as being eligible for participation based on predefined in- and exclusion criteria and were randomized into the study (Fig. 1). Of the 161 participants randomized into the study, 151 completed all study visits. Reasons for withdrawing early from the study included withdrawing at the beginning of the run-in phase (*n* = 2), illness (*n* = 6), smoking (*n* = 1), and family circumstances (*n* = 1).

Of the 151 study participants who completed all study visits, 138 were considered as being compliant with the study protocol, and hence, included in the PP analysis. In contrast, 13 participants (9%) were excluded for reasons including low compliance to test product intake (i.e., <90% compliance, *n* = 3); use of concomitant medications that might have affected study outcomes (*n* = 6); recorded a weight change of >5% over the study period (*n* = 2), and having missing blood samples (*n* = 2). A sub-group of 39 participants with established T2DM completed the mixed meal test and their data was included in the analysis of the postprandial outcomes.

An overview of the study participants’ characteristics at baseline is provided in Table 2. There were no significant differences in baseline characteristics such as age, gender, BMI, blood lipids, fasting plasma glucose, and hemoglobin A1c (HbA1c) between the two groups. Of the 138 participants included, 59 were women (42.8%) and 79 were men (57.2%); 44 (31.9%) had established T2DM and 94 (68.1%) were at high-risk of developing T2DM. They had baseline TG concentrations ranging from 1.7 to 9.0 mmol/l and LDL-C concentrations ranging from 1.8 to 7.3 mmol/l.

**Table 2:** Baseline characteristics of study participants (*n* = 138)

Characteristics	Placebo, *n* = 70	PS intervention, *n* = 68
Age (years)	58.3 ± 10.0^a^	58.7 ± 10.4
Gender	40 males, 30 females	39 males, 29 females
T2DM status		
Established T2DM	*n* = 22	*n* = 22
At risk of T2DM	*n* = 48	*n* = 46
BMI (kg/m^2^)	33.7 ± 5.7	32.5 ± 4.8
Statin use		
Established T2DM		
Yes	11 (15.7%)	11 (16.2%)
No	11 (15.7%)	11 (16.2%)
At risk of T2DM		
Yes	6 (8.6%)	7 (10.3%)
No	42 (60.0%)	39 (57.3%)
Use of hypoglycemic medication		
Established T2DM		
Yes	18 (25.7%)	16 (23.5%)
No	4 (5.7%)	6 (8.8%)
At risk of T2DM		
Yes	1 (1.4%)	0 (0.0%)
No	47 (67.1%)	46 (67.7%)
TG, mmol/l	2.41 ± 0.79	2.50 ± 0.86
Total cholesterol, mmol/l	5.73 ± 1.09	5.78 ± 1.18
LDL-C, mmol/l	3.81 ± 0.97	3.81 ± 1.02
HDL-C, mmol/l	1.18 ± 0.27	1.20 ± 0.32
Glucose, mmol/l		
Established T2DM	8.42 ± 1.78	8.54 ± 1.67
At risk of T2DM	5.81 ± 0.55	5.99 ± 0.63
HbA1c, %^a^		
Established T2DM	6.88 ± 0.95	7.02 ± 1.05
At risk of T2DM	5.39 ± 0.40	5.44 ± 0.42

Mean ± standard deviation (SD); all such values

There were no statistically significant differences in baseline characteristics between the placebo and intervention groups

*T2DM* type 2 diabetes mellitus, *TG* triglycerides, *LDL-C* low-density lipoprotein cholesterol, *HDL-C* high-density lipoprotein cholesterol, *HbA1c* hemoglobin A1c

^a^HbA1c was measured at screening

While individuals with established T2DM and at risk of developing T2DM did not differ in their baseline TG concentrations (2.41 ± 0.88 with T2DM vs. 2.48 ± 1.13 mmol/l at risk of T2DM), TC and LDL-C concentrations were different. Individuals with T2DM had lower TC (5.24 ± 1.12 mmol/l) and lower LDL-C (3.49 ± 0.89 mmol/l) than those at risk of developing T2DM (TC: 6.14 ± 1.06 mmol/l; LDL-C: 4.17 ± 0.84 mmol/l). This can be explained by statin use. Of the 44 individuals with T2DM, 22 were using statins, while of the 94 individuals at risk of developing T2DM only 13 were on statin therapy. Statin users were taking 10–20 mg/d atorvastatin, 20–40 mg/d simvastatin, 40 mg/d pravastatin or 5–20 mg/d rosuvastatin. Those using statins had lower baseline cholesterol concentrations. Individuals with established T2DM had baseline glucose and HbA1c concentrations of 7.89 ± 1.83 mmol/l and 6.95 ± 0.99 %, respectively, as compared to 5.38 ± 0.66 mmol/l and 5.40 ± 0.41 % in individuals at risk of developing T2DM. Body weights of the study population were 96.2 ± 18.0 kg (median 95.4 kg) at baseline and 96.0 ± 18.0 kg (median 95.7 kg) after 6 weeks PS intake with no statistically significant difference between the placebo and PS intervention group.

### Adverse events

A total of 143 AEs were reported throughout the study, of which 96% (*n* = 137) were accounted as non-serious AEs. One AE was scored ‘definitely related’ to the study product (i.e., loose stools), six were scored as being ‘possibly related’ and 14 were scored as ‘unlikely to be related’. The remaining 116 of the 137 non-serious AEs were scored as being ‘not related’ to the study product. There were six serious AE (SAEs) reported throughout the study. Two were scored as being ‘unlikely to be related’ to the study product and the remaining four were scored as ‘not related’ to the study product.

### Fasting blood lipids and glucose and insulin

Intake of the PS-added spread for 6 weeks lowered TG concentrations by 0.20 ± 0.09 mmol/l (−8.3%) as compared to control spread intake (Table 3). Sub-group analysis defined by having a baseline TG below and above the median baseline TG concentration of 2.25 mmol/l resulted in a −8.1% ((95% CI 2.3 to −17.4%) in those below and −8.6% (95% CI 1.7 to −17.8%) in those above the median, hence not different from the overall −8.3% effect. LDL-C decreased by 0.18 ± 0.07 mmol/l (−4.6%) and TC concentrations by 0.24 ± 0.09 mmol/l (−4.2%). None of the study participants had a LDL-C concentration of <1.8 mmol/l at baseline or post intervention. Regarding a LDL-C target of <2.6 mmol/l, 9 (6.5%) individuals, four in the placebo and five in the PS intervention group, had a LDL-cholesterol <2.6 mmol/l. After 6-weeks PS intake, 13 (9.4%) individuals, five in the placebo and eight in the PS intervention group, had a LDL-cholesterol < 2.6 mmol/l. No significant changes were found for HDL-C, non-HDL-C, remnant C, and Lp(a) concentrations, or for glucose and insulin concentrations, between the intervention and placebo group. There were further no differences or interaction effects of gender, BMI, T2DM status (established vs. at risk) or study site (Sydney vs. Adelaide).

**Table 3:** Fasting blood lipid and glucose and insulin concentrations at the end of the 6-week intervention period

	End of intervention		Relative (%) difference vs placebo (95% CI)	*p*-value
Parameter	Placebo LS-means^a^ (95% CI)	PS intervention LS-means (95% CI)		
TG, mmol/l	2.40 (2.27 to 2.53)	2.20 (2.08 to 2.32)	−8.3 (−1.1 to −15.0)	0.024
TC, mmol/l	5.70 (5.58 to 5.83)	5.46 (5.35 to 5.58)	−4.2 (−1.2 to −7.1)	0.006
LDL-C, mmol/l	3.81 (3.71 to 3.90)	3.63 (3.54 to 3.72)	−4.6 (−1.2 to −8.0)	0.009
HDL-C, mmol/l	1.15 (1.13 to 1.17)	1.15 (1.13 to 1.17)	0.2 (2.6 to −2.1)	0.877
Non-HDL-C, mmol/l	4.50 (4.38 to 4.62)	4.28 (4.17 to 4.39	−4.8 (−1.2 to −8.4)	0.010
Remnant-C, mmol/l	0.65 (0.62 to 0.70)	0.60 (0.57 to 0.64)	−7.6 (0.9 to −15.3)	0.078
Lp(a), nmol/l	27.85 (26.74 to 28.99)	27.71 (26.59 to 28.88)	−0.5 (5.4 to −6.1)	0.866
Glucose, mmol/l	6.57 (6.46 to 6.69)	6.57 (6.46 to 6.69)	0.0 (2.5 to −2.5)	0.999
Insulin, mU/l	11.69 (11.02 to 12.40)	11.61 (10.94 to 12.33)	−0.6 (8.1 to −8.6)	0.880

^a^LS-means were based on the model with the smallest BIC

*TC* total cholesterol, *TG* triglycerides, *LDL-C* low-density lipoprotein cholesterol, *HDL-C* high-density lipoprotein cholesterol, *non-HDL-C* non high-density lipoprotein cholesterol, *remnant-C* remnant cholesterol, *Lp(a)* lipoprotein (a)

### Postprandial blood lipids, glucose and insulin

The blood lipid characteristics of the 39 individuals aged 62.0 ± 8.7 years who completed the postprandial challenge differed in their TC, TG, and LDL-C concentrations at the end of 6-week intervention and the start of the postprandial challenge (average value of blood samples at −15 min and −5 min defined as baseline). These individuals were randomly selected and hence not matched for their baseline concentration. To control for the difference in concentrations at the start of the postprandial test, the postprandial responses are depicted as corrected for baseline.

**Fig. 2:**
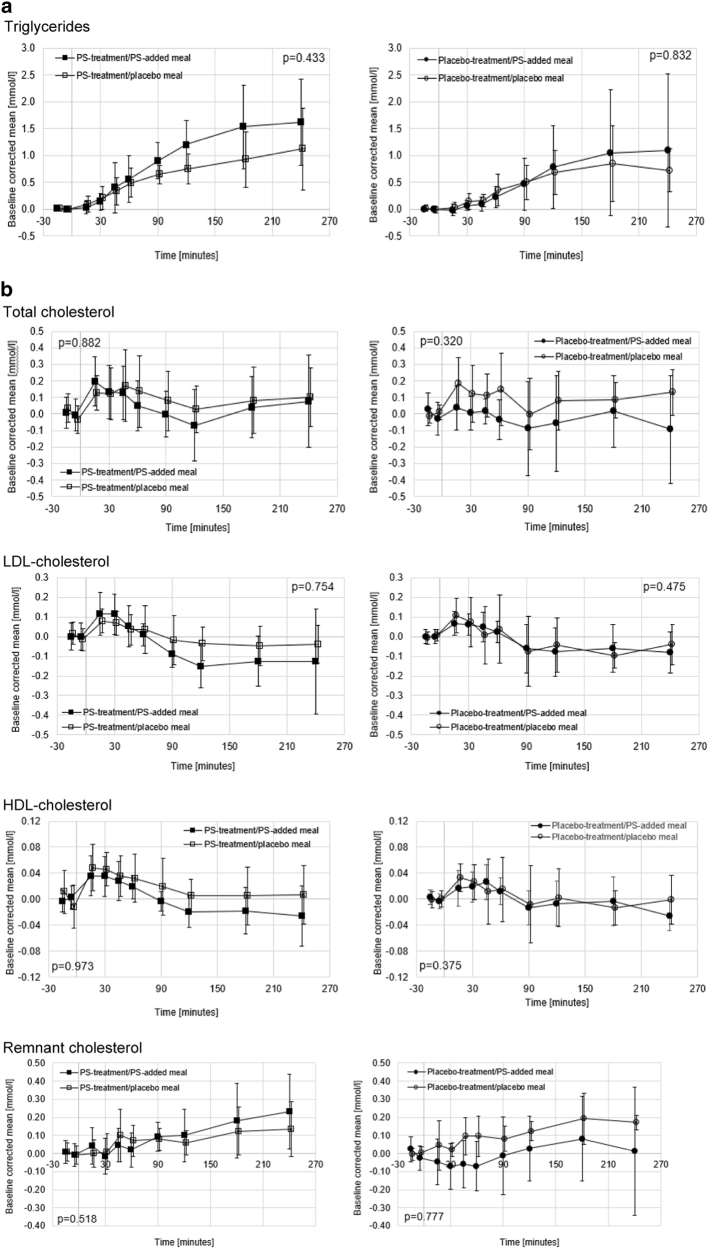
**a** Postprandial response of plant sterols on triglyceride (TG) concentrations (data are mean ± SD). **b** Postprandial response of plant sterols on total cholesterol, LDL-C, HDL-C, and remnant cholesterol concentrations (mean ± SD). Test for shape difference of the type of meal challenge was non-significant for all blood lipid parameters (*p* values as indicated). The number of individuals in the four groups were: PS intervention group and either test meal without added PS (*n* = 11) or test meal with added PS (*n* = 11); control group and either test meal without added PS (*n* = 10) or test meal with added PS (*n* = 7)

No effects of acute or chronic PS intake on the shape of the postprandial response for blood TG concentrations were found (Fig. 2a). Blood TG concentrations steadily increased over time reaching a plateau at around 240 min but not yet returning to baseline values (Fig. 2a). Based on non-baseline corrected curves, the TG concentrations were about 30–35% higher (*p* = 0.011) in individuals who had 6-week PS treatment as compared to placebo treatment. The response over time after the consumption of the challenge meal for TC, LDL-C, HDL-C, and calculated remnant C concentrations is depicted in Fig. 2b. LDL-C increased above baseline during 30 min after consuming the challenge meal and returned to baseline thereafter. Remnant cholesterol increased after 45 min steadily until 240 min. It appeared that remnant cholesterol concentrations were 28% higher in individuals who had the 6-week PS treatment as compared to the placebo treatment (*p* = 0.045).

**Fig. 3:**
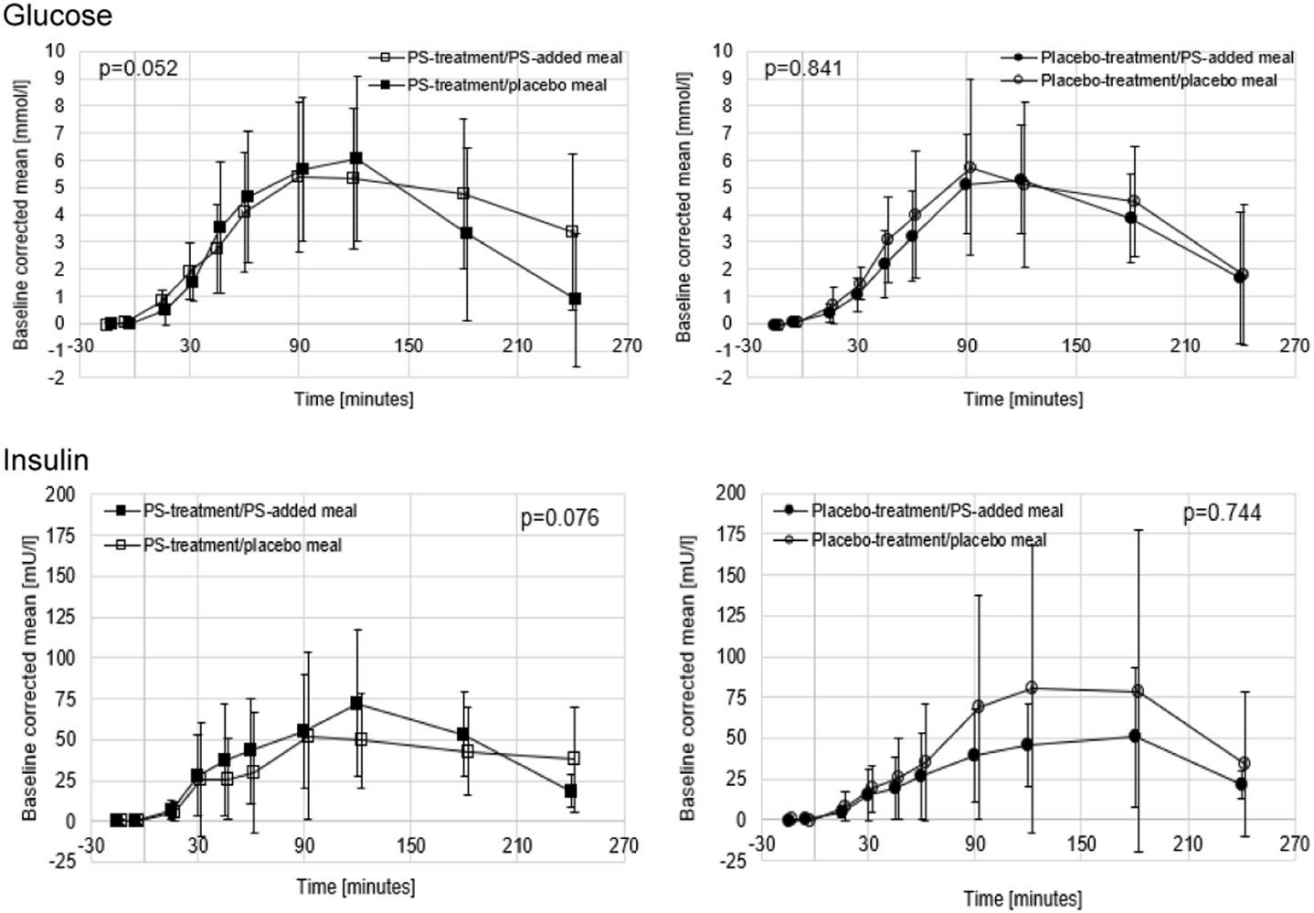
Postprandial response of plant sterols on glucose and insulin concentrations (mean ± SD). Test for shape difference of the type of meal challenge was non-significant for glucose and insulin (*p* values as indicated). The number of individuals in the four groups were: PS intervention group and either test meal without added PS (*n* = 11) or test meal with added PS (*n* = 11); control group and either test meal without added PS (*n* = 10) or test meal with added PS (*n* = 7)

Postprandial glucose and insulin concentrations increased up to 120 min after consumption of the meal challenge and had not fully returned to baseline after 240 min (Fig. 3). No effects of acute or chronic PS intake on postprandial glucose and insulin concentrations were found neither for the curve shapes (Fig. 3) not for tAUC for insulin and the piAUC for glucose as measured over 120, 180, and 240 min.

## Discussion

This randomized controlled study showed a significant reduction in both blood TG and LDL-C concentrations following 6-week intake of a spread with 2 g/d of PS as compared to a control spread, demonstrating that PS have a dual blood lipid benefit in individuals at risk of developing or with established T2DM.

The observed reduction in TG concentrations with an absolute change of 0.20 mmol/l or a relative change of 8.3% in individuals at risk of or with established T2DM, and with baseline TG concentrations of 2.5 ± 0.8 (range 1.7 to 6.0 mmol/l), is in line with TG lowering effects described in the meta-analysis of Demonty et al.^12^. Considering a baseline TG concentration of 1.90 mmol/l, a reduction in TG of −0.18 mmol/l; range −0.27, −0.10 was found^12^. This further demonstrates that PS lower TG concentrations, especially in individuals with high basal TG concentrations^12,18^.

Combining results from five studies including a total of 127 patients with T2DM consuming an average daily dose of 1.85 g PS or plant stanols, Plat et al.^10^, indicated that TG concentrations were lowered by an average −0.06 mmol/l (3.7%). Notably, in these studies, individuals were not selected based on having elevated TG concentrations, which may explain the much lower TG lowering effect found in these diabetic study populations as compared to our finding. In contrast, based on four studies with a total of 185 individuals with the Metabolic Syndrome who consumed an average intake of 3.24 g/d of PS or plant stanols, TG were lowered by −0.66 mmol/l or 13.9%^10^. Basal TG concentrations exceeded 1.7 mmol/l in this Metabolic Syndrome study population which is comparable to the population investigated in this study. In the present study, no differences in blood TG responses between individuals with established T2DM compared to those at risk of developing T2DM were found. This further supports that elevated baseline TG concentrations (2.48 ± 1.13 mmol/l for those at risk of T2DM and 2.41 ± 0.88 mmol/l for those with established T2DM) are responsible for the observed lowering of TG concentrations rather than the health or diabetic status of individuals as such.

Consistent with findings from various meta-analyses^9,11^, this study again demonstrates that an intake of 2 g/d of PS lowers TC and especially LDL-C and non-HDL-C concentrations. The magnitude of change in LDL-C, i.e. the 4.6% lowering seems to be on the lower end of what one would have expected for a daily PS intake of 2 g. Nevertheless, the 95% CI (−8.0 to −1.2%) partly overlaps with those reported in the meta-analysis by Ras et al.^11^. Based on studies performed in T2DM patients, Plat et al.^10^ concluded that a large inter-individual variability in the extent of LDL-C lowering exists. At an average intake of 1.85 g/d of PS and plant stanols, the average LDL-C reduction in T2DM patients was 0.23 mmol/l or −6.3%. This finding is consistent with the average LDL-C effect of 0.18 mmol/l or −4.6% observed in our overall study population.

Patients with T2DM and those with the Metabolic Syndrome, who are at increased risk of developing T2DM, are known of having a high cholesterol synthesis efficiency coupled with a low intestinal cholesterol absorption efficiency^19,20^. A reason for this variation in cholesterol metabolism is related to altered insulin resistance suggesting that better insulin sensitivity is linked to a higher cholesterol absorption^19^. Furthermore, it was also shown that body weight regulated cholesterol metabolism in T2DM patients and that with increasing body weight and BMI, cholesterol absorption was low and cholesterol synthesis high^21^. Whether indeed individual variation in cholesterol absorption/synthesis efficiency due to the degree of obesity and insulin resistance explains the variability in the LDL-C lowering response and the overall lower effect remains speculative. Nevertheless, a potential association with blood glucose concentrations and/or insulin resistance on the responsiveness to cholesterol-lowering interventions that affect intestinal cholesterol absorption such as PS and stanols has been reported^15,22^. For instance, de Smet et al.^15^ found that healthy individuals who had a more pronounced basal postprandial glucose response experienced a smaller LDL-C lowering after 3 weeks of plant stanol intake.

In the present study, there was however no evidence that the treatment effect could be differentiated by gender, BMI and by diabetes status; in other words, those at risk of developing T2DM showed the same benefits as those with established T2DM. No effects of PS intervention were found for Lp(a) concentrations. This is not unexpected since so far, no dietary interventions have shown a meaningful effect on lowering Lp(a) including at least three studies that showed PS or plant stanol interventions have no effect on Lp(a)^23–25^. PS intervention had further no effect on fasting blood glucose and insulin concentrations, which is in line with previous evidence in T2DM patients^26,27^.

Regarding the postprandial responses, no differential effects were found for postprandial TG, TC, LDL-C, HDL-C, and remnant C and the postprandial response of glucose and insulin. However, a substantial variability between individuals was seen with both the acute and chronic meal challenge which could possibly be as the limited number of participants a reason for not seeing statistically different effects in postprandial responses. The finding that individuals, who chronically consumed the PS added spread for six weeks, had higher absolute concentrations in their postprandial TG responses, suggests that liver driven mechanisms may have contributed to these subtle differences. There is increasing evidence suggesting that particularly elevated postprandial TG may be an early feature of dysregulation in glycemic control and may even be predictive of the development of T2DM as well as atherosclerosis over time^14,28^. Nevertheless, two recent studies measuring postprandial responses after a meal challenge containing 3 g plant stanols^16^ or 4 g PS or stanols^15^ also did not find any effects on postprandial TG concentrations during 4 to 8 h in healthy individuals. In these studies, baseline TG concentrations of study participants were in a healthy range (1.1–1.3 mol/L)^15,16^) as compared with elevated basal TG concentrations of 2.5 mmol/l of study participants in this study. Other studies relying on different postprandial study designs also did not find an effect of plant stanols on postprandial cholesterol and TG concentrations after a high- or low-fat breakfast^29–31^. Whether adding just 2 g PS to the challenge meal instead of higher amounts as used in the previous studies and using a 4.2 MJ test meal as compared to 3.2–3.3 MJ used in other studies may have impacted the outcome of the postprandial response remains conjectural.

While the (LDL-) cholesterol lowering effect of PS or stanols is known to result from the inhibition of intestinal cholesterol absorption^32^, the underlying mechanism by which they lower TG concentrations is still not well understood^18^. Considering the lack of effect on postprandial lipids, especially TG, raises the question about the mechanism(s) that causes the observed lowering of fasting TG concentrations after PS intake. Based on mostly animal study evidence, it is suggested that PS or stanols may lower circulating TG concentrations via multiple mechanisms, including intestinal derived, i.e. absorption site effects like reduced intestinal TG and fatty acid (FA) absorption and increased fecal FA excretion^18^. There is further evidence that PS and stanols affect hepatic FA and TG metabolism^18^. However, human studies investigating postprandial fat handling in normo-triglyceridemic individuals have not found that PS or stanols interfere with intestinal fat digestion and absorption^29,30^. This may explain the lack of an effect of PS on postprandial TG as well as on postprandial cholesterol carried in different lipoproteins. It was hypothesized that the fasting TG lowering mechanism of PS is of hepatic origin based on a reduction in hepatic very-low density lipoprotein (VLDL) secretion reported in mice fed a PS or stanol enriched diet^33^ and a reduction in circulating (large and medium size) VLDL particles after consuming 2 g/d of plant stanols in dyslipidemic individuals with the Metabolic Syndrome^34^. However, the concept that PS may reduce hepatic TG secretion can be challenged as Nakajima et al^35^ demonstrated that hepatically derived TG-rich lipoproteins, i.e. VLDL and VLDL remnants account for about 80% of the increase in postprandial TG.

A limitation of this study is that the postprandial responses were only studied in a subgroup and only for 4 h after consuming the challenge meal. Clearly, postprandial TG did not return to baseline after 4 h and therefore a longer time, e.g., 6 h would have been more desirable, revealing the effect of PS on TG catabolism in the late postprandial stage. Another limitation is that we did not measure serum apolipoprotein (apo) concentrations either in the fasted or postprandial state. Such data could have given some more insights into possible underlying mechanisms, especially related to apo B carrying lipoprotein particles. A strength of this randomized controlled study is that we included a large number of individuals who were followed up under well-controlled, double-blind conditions making this the first PS intervention study that enrolled individuals at risk of and with established T2DM, and who also had both elevated baseline LDL-C and TG concentrations.

The rise in the global prevalence of T2DM is metabolically associated with dyslipidemia and an increased risk of CVD. Therefore, the dual lipid lowering effect of PS could be of great benefit for individuals with or at risk of T2DM. LDL-C is already recognized as a causal risk factor for arteriosclerotic CVD^36^ and a reduction in LDL-C concentration as achievable with PS intake would be expected to contribute to reducing the risk of CVD. Moreover, evidence has emerged that elevated TG concentrations are also an independent risk factor for CVD with a modest association between elevated fasting TG concentrations and increased CVD risk^37^. A meta-analysis of prospective studies concluded that per 1.0 mmol/l increase in fasting TG, the risk of major cardiovascular events increases by 27%^38^. Human genetic studies further support a causal relationship between TG and CVD risk^39^.

In conclusion, this study demonstrates that 2 g/d of PS can lead to a dual benefit of lowering TG and LDL-C concentrations in individuals at risk of and with established T2DM as well as elevated TG and LDL-C concentrations, and even when taking statins. No effects were found on HDL-C, non-HDL-C, remnant C, fasting glucose and insulin, and on postprandial blood lipids and glycemic response after acute and chronic PS intake. The TG next to the LDL-C lowering benefit of PS can be considered of clinical relevance and may help to further reduce the risk of CVD as part of a healthy diet and lifestyle approach.
